# ‘I want to know why and need to be involved in my own care…’*:* a qualitative interview study with liver, bile duct or pancreatic cancer patients about their experiences with involvement in care

**DOI:** 10.1007/s00520-018-4548-8

**Published:** 2018-11-14

**Authors:** Farzana Ibrahim, Per Sandström, Bergthor Björnsson, Anna Lindhoff Larsson, Jenny Drott

**Affiliations:** 1 Department of Clinical and Experimental Medicine, Department of Surgery, County Council of Östergötland, Linköping University, Linköping, Sweden; 2 Department of Medicine and Health Sciences, Division of Nursing Science, Linköping University, Linköping, Sweden; 3 Faculty of Medicine and Health Sciences, Linköping University, 581 83 Linköping, Sweden

**Keywords:** Upper abdominal cancer, Involvement, Qualitative research, Surgery, Fast-track care programme

## Abstract

**Purpose:**

Patients’ involvement in their own care is important for those with upper abdominal tumours. Care is often conducted according to standardized fast-track care programs (FTCP), and a shorter hospital stay is one of the goals. However, there is no research providing an in-depth perspective on patients’ experiences of involvement in care. In this qualitative study, we explored experiences of involvement among patients who had surgery for upper abdominal tumours and were cared for according to an FTCP.

**Methods:**

Qualitative in-depth face-to-face interviews about patient involvement in care were conducted with 20 patients who had surgery for the liver, bile duct, or pancreatic cancer using an open-interview guide.

**Results:**

The most important findings are that customized information and active dialogue about care decisions stimulate patient involvement. We identified three themes from the analysed data: involvement depended on the quality of information, communication and involvement during the care period, and safety at discharge.

**Conclusions:**

Individualized care and continuous information about treatment and care goals in the FTCP during the care process create trust between patients and healthcare professionals and increase patient experiences of involvement.

## Introduction

Patients with tumours in the liver, bile ducts and pancreas often need to make difficult treatment decisions. After investigation and diagnosis, patients are informed about the possibility of surgical treatment that is based on their overall health condition, as well as on tumour-specific factors. These patients often have a major surgical procedure to go through, leading to a long period of recovery [1–4]. The care of patients with upper abdominal malignancies is being carried out to an increasing extent according to a fast-track care programme (FTCP), and the care team follows a standardized programme that includes a number of actions; compared to traditional care, evidence shows these actions reduce postoperative complications [5]. A shorter hospital stay means that patients will have a longer recovery period at home. Collaborative care and patient involvement are important to achieve the care goals in the FTCP. However, beyond achieving these goals, it is also necessary that patients have the ability to leave the hospital earlier. Patient involvement or participation in care promotes patient satisfaction and is considered a basic condition for good care. Patient participation has been used interchangeably with the concepts patient empowerment and patient-centeredness. A concept analysis by Castro et al. [6] has analysed the different definitions of the close concepts; and by embracing patient participation as a strategy, care can improve patient-centeredness, which will facilitate patient empowerment [6]. Patient participation is a complex concept and is also closely related to patient safety [7]. Earlier research on patients with chronic diseases has shown that patients’ involvement in their own care is associated with improved treatment outcomes, rehabilitation and recovery [8].

To our knowledge, research is lacking in patients undergoing surgery for upper abdominal cancers concerning their experiences of involvement in care. Previous research has mainly focused on treatment decision-making and health-related quality of life [9–11]. Shared decision-making improves care and ensures that the patient is at the centre of the care. The level of involvement and medical decisions are based on the patients’ preferences and needs and on discussions about existing scientific evidence, together with the expertise of healthcare professionals [12]. Cancer care is often complex and requires flexibility. Patients with cancer often need to face difficult decisions that may influence their health from both the short- and long-term perspectives [13].

Knowledge about experiences of involvement may improve tailored care for patients with surgery for upper abdominal cancers. However, there is no research focused on the in-depth perspective of patients’ experiences of involvement in care. In this qualitative study, we aim to explore patient experiences of involvement in FTCP services received after surgery for upper abdominal tumours.

## Methods

### Participants and sampling

This qualitative study included patients from a university hospital and was a single-centre study. The study was a part of a larger project about patient participation in surgical care with both qualitative and quantitative methods. This qualitative study was initiated and designed by the last author (JD).

All patients were informed about the study by the interviewer, and written informed consent in line with the Declaration of Helsinki (WMA 2013) was obtained. This study was approved by the Research Ethics Committee in Linköping, Sweden (No. 2016/276–31).

Inclusion criteria were patients who had surgery for the liver, bile duct or pancreatic cancer; spoke the Swedish language; were at least 18 years of age and received care according to the FTCP. Overview of the components included in the standardized fast-track care programme is illustrated in Table 1. All 20 included patients were recruited from November 2016 to December 2017 at a specialist surgical clinic in Sweden. Table 2 summarizes the patient characteristics of the interviewees at the time of the interview.

**Table 1:** Overview of the components in the standardized fast-track care programme

Phases	Fast-track care components
Preadmission and preoperative	Smoking and alcohol cessation
	Nutritional support
	Medical optimization
	Patient preoperative information
	Preoperative fasting and preoperative carbohydrate fluids
	Thrombosis prophylaxis
	Skin preparation-infection prophylaxis
	Nausea prophylaxis
Intraoperative	Maintain fluid balance
	Restrictive use of drains
	Remove nasogastric tubes in OR, no routine use
	Standardized anesthesia with low-dose opioids or patient-controlled epidural
Postoperative	Early mobilization
	Early intake of fluids (oral) and removal of intravenous fluids
	Early removal of urinary catheters
	Nutritional supplements
	Glucose control
	Multimodal approach to control pain and nausea/vomiting
	Early discharge

*OR*, operating room

**Table 2:** Patient characteristics of interviewees at the time of interview

Patient	Age	Sex	Social status	Diagnosis, malignancy
1	68	Male	Life partner	Bile duct
2	64	Female	Life partner	Liver
3	77	Male	Life partner	Liver and bile duct
4	54	Male	Living alone	Liver
5	66	Male	Life partner	Liver
6	63	Female	Life partner	Bile duct
7	74	Female	Life partner	Pancreatic
8	83	Male	Life partner	Pancreatic
9	63	Female	Life partner	Bile duct
10	70	Female	Life partner	Liver
11	66	Female	Life partner	Liver
12	78	Male	Life partner	Pancreatic
13	77	Female	Living alone	Pancreatic
14	54	Male	Life partner	Liver and bile duct
15	79	Female	Life partner	Bile duct
16	65	Male	Life partner	Pancreatic
17	55	Male	Living alone	Bile duct
18	75	Female	Living alone	Pancreatic
19	70	Male	Life partner	Pancreatic
20	56	Female	Life partner	Pancreatic

The sampling strategy was purposeful, and we aimed for a maximum variation of sex, age and cancer diagnoses. An interview guide about involvement that included open-ended questions was used, developed by the research team and based on existing literature [14]. The interview guide was pilot tested in one patient to ensure the clarity; no adjustments were made. Open-ended, in-depth face-to-face interviews were conducted by the first author (FI), a specialist nurse in surgical care. The interview meeting was booked at a location decided by the patient. Before the interview began, a relaxed atmosphere was created so that the patients would feel safe and comfortable. All interviews started with a question on the patients’ experiences and perspectives regarding their involvement in relation to their surgical care. Probing and looping questions were used continuously during the interviews in relation to the patients’ specific experiences of involvement. Interview areas included follow-up questions and clarifications about involvement and possibility to influence the care and decisions taken during the care period [14]. The time between surgery and the interview ranged from 2 to 9 months (median 5 months), and the interviews lasted between 15 and 45 min (median 26 min).

### Data analysis

The interviews were transcribed verbatim by the first author; the data analyses were processed and followed the six phases of thematic analysis according to Braun and Clarke [15]. The data analysis started with familiarization, and the transcribed texts were read several times. Initial thoughts were noted and initial codes generated. Codes were identified according to the study aim, and the next step was to search for themes. All codes relevant to the research aim were incorporated into a theme, and these larger sections of data defined the themes. The procedure to conduct the thematic analysis according to Braun and Clarke and meet the trustworthiness criteria in this study were a reflective process moving back and forward between the six phases [14–16]. The analysis followed (1) familiarization with the data, (2) generating initial codes, (3) searching for themes, (4) reviewing themes, (5) defining and naming themes and (6) producing the report [15, 16]. The six phases establishing trustworthiness during each phase of thematic analysis and included to, e.g., document thoughts about themes, peer debriefing, researcher triangulation and description of the audit trail [16]. Discussions among the research team about the findings were carried out in order to increase the trustworthiness of the results and prevent interpretation bias. The four criteria by Lincoln and Guba for establishing trustworthiness in qualitative studies were also discussed thru the research process: credibility, dependability, confirmability and transferability [17]. The sampling strategy was purposeful, and we aimed for a maximum variation of sex, age and cancer diagnoses to obtain a variety of experiences to enhance credibility. The data represents the reality, and the findings are consistent and strengthened by peer-debriefing (dependability). The findings are not an outcome of the subjectivity or bias of the researchers, and the used quotations illustrated the patients’ words (confirmability). To make this study results transferable, descriptive information about the included patients was presented, to make it possible for the reader to evaluate the findings to other contexts.

## Results

The most important finding is that customized information and active dialogue about care decisions stimulate patient involvement. We identified three themes from the analysed data: involvement depended on the quality of information, communication and involvement during the care period and safety at discharge.

### Involvement depended on the quality of information

The patients felt that they had received helpful information from surgeons preoperatively. The patients had been informed of the diagnosis and the surgical procedure as well as about the entire treatment process. They were satisfied with the preoperative information. When the doctor took the time and explained and answered all questions, patients felt confident and involved. Patients received much information preoperatively, though they thought it was too much in one single meeting. The patients had trouble concentrating, and several of them could not remember whether they had received information about the care process or not. They thought it would have been better if the information was divided into several occasions, and they were also missing written information about the FTCP preoperatively.

Patient: ... There was so much information at one time ... I had just received word that I had cancer and also about the poor prognosis, and I tried to concentrate on anything they said; it wasn’t easy ... they should not give more information; it is better to send written information so you can read or to come back another day and get the information about the actual healthcare. (P 9)

In view of the fact that the patients could not remember all the information about the progress of postoperative care, they felt uncertain about what healthcare-related activities were scheduled during the postoperative period. The experience of many patients was that of not always receiving the information about care activities or decisions to be made during the postoperative care period. Health professionals did not always inform them why some action was performed, and the patients did not always understand why various activities were carried out. All patients needed more detailed information during their hospital stay to feel more involved in their care.

Patient: ... I lacked information ... You must get up! And I did, I got up and ate and did everything that the staff said ... and of course, I knew it was good, but there was no one who told me the reason. (P 16)

All of the patients related their experiences of involvement with information. Patients indicated the need to obtain customized information and gain knowledge about what to do and why. It was also felt that the communication between healthcare professionals and the patient was important. When professionals were responsive, showed respect and communicated with the patient, patients felt themselves worthier and felt that they were a part of the team. Patients with special needs had not perceived that the healthcare providers understood their information needs. They pointed out the need for repeated information and more time to explain the information.

Patient: ... I got the information ... well, I am dyslexic, so I had asked for information before surgery. I wanted oral and written information ... but it was not possible, and it was stressful for me ... They could take the time and make a call, just to tell and give a brief information. (P 4)

Dissatisfaction resulted among some patients when they had not received enough information about possible postoperative complications. The consequence was that patients felt fear and were unprepared for what happened. They felt that if they had been properly informed, they would not be fearful or end up in a precarious situation.

Patient: What I thought was bad was that they had not informed me that I could get diabetes after surgery, that the pancreas had ceased to produce insulin ... I was totally unprepared, it came as a complete surprise ...I had wanted to be forewarned. (P 8)

Despite unfulfilled wishes, as mentioned above, patients who received information about the postoperative care process felt secure. They experienced that they were involved in their care and that the staff explained in advance the different care stages that were to be carried out. This helped patients be prepared for what would come next, and it brought a sense of security and involvement.

### Communication and involvement during the care period

The patients felt that communication between healthcare professionals and the patients was of major importance. When healthcare professionals came with different options regarding care or treatment and allowed patients to be involved, patients became automatically involved and felt they were one of the team. Although communication was seen as an important part of the process, it was even more important that professionals did not use a medical jargon.

Patient: ... You didn’t know why they did certain things ... somewhere I feel that they forget it is a little human being there, which needs to understand and pose its questions and get answers ... they must always know that is a new situation for every unique patient, and all patients are different. (P 9)

Among the patients who sought the opportunity to be involved in decisions about their care, there were patients who argued that it was easier to entrust the decision to doctors and health care professionals. They argued that the healthcare professionals had more knowledge and that they had reasons for the different decisions. When it was the patients’ experience that they had insufficient knowledge, they became passive and followed the healthcare professionals’ directive.

### Safety at discharge

Postoperatively, when patients were discharged, they had different experiences depending on how prepared they were. Sometimes, the discharge had been planned in advance, and the patient had been well prepared. Other times, the decision was made quickly because of various reasons (e.g. lack of space on the ward). The patients who had prepared for the discharge felt a sense of safety. They had received answers to their questions as well as information about return visits to the outpatient clinic and about their treatment plan. The patients who were not prepared for discharge were afraid and anxious; this was especially the experience of patients who were living alone. Sometimes, patients were discharged due to shortages of staff or space in the hospital. Patients felt that they needed to take responsibility for the staff shortage and the stressful situation at the hospital. Other patients who did not feel ready for discharge felt that they had been neglected.

Patient: ... They only say ... now you have to go home because we have a shortage of beds in the hospital ... I do not care, I said. I am not going home until I feel better and can eat; otherwise I don’t go home, I said. (P 16)

Some of the patients came back to the hospitals due to various reasons, including that they were not in a condition of good health when they were discharged. Patients who had received contact information for the healthcare team and had scheduled care planning felt safe before going home. They had a positive experience, and all of them had a sense of security.

Patient: I felt very confident in going home ... I had a care planning meeting at home, and I felt very safe… (P 17)

## Discussion

This is, to our knowledge, the first qualitative study focusing on patient involvement in patients who have had surgery for the liver, bile duct or pancreatic cancer and received fast-track care. The most important finding in this study shows that involvement depended on the quality of information and communication about care decisions stimulates patient involvement. Care according to an FTCP includes early mobilization and nutritional intake, leads to enhanced recovery in both the short and long term and benefits the patients. The quality of care improves, and a shortened hospital stay reduces costs [18]. When patients are not getting enough information about what FTCP means, they do not understand the ideas behind the various actions carried out during the postoperative course.

A study by Aasa et al. [19] shows that healthcare professionals must provide more information to patients throughout the care period to enable their participation and encourage them to take responsibility [19]. Healthcare professionals should inform the patients about the responsibility that they have regarding their own recovery, thus increasing patient involvement.

Repeated information with quality stimulates patient involvement, and it also promotes the health of patients. Nonetheless, it is still a challenge to customize information and involve patients in their own care and in decision-making processes [20]. Patients also experienced the stressful environment for the healthcare professionals and the lack of staff time to answer their questions and give individualized information. Because all patients are different and have different healthcare needs, it is important that care, despite FTCP, is flexible for each individual patient and his or her needs. Patient involvement as a strategy, can improve patient-centeredness and also improves patient health [6, 21].

Several patients in this study felt that it was easier to let the staff make health-related decisions because they were the experts. The patients felt that the doctors had knowledge and, as patients, they would not question their decisions. In these situations, patients adopted a passive role. This may result from not having enough customized information and involvement. Other studies about decisional conflicts, such as lack of information and clarity, have similarities with our findings [22, 23].

Due to the last theme about safety at discharge, patients should not have to take responsibility for or be sent home due to a lack of staff or hospital beds. Patients who have had major surgery for the liver, bile duct or pancreatic cancer should get the care they need without having to claim their rights. Individualized repeated information may reduce anxiety and readmissions and result in a safe environment at discharge. It needs to be acknowledged that patients experienced this to be of importance in order to enter surgical care with a positive mind set. Previous evidence has suggested that patients who had cancer surgery and a positive mind set had better outcomes [24]. Castro et al. 2016 described a positive attitude as an antecedents and also patient information and a supportive care environment as an empirical referents of patient participation [6]. Professionals must have insight into patients’ insecurities to encourage the patients’ sense of involvement. The fast-track care model has been shown to reduce both the length of hospital stay and the postoperative recovery time of patients compared to traditional care [25], but more research is needed. The findings of the current study suggest a need for discharge monitoring, especially in those patients who were discharged from the hospital before they believed they were ready. In the immediate post-discharge period, patients often feel vulnerable, and intensive discharge monitoring may prevent unnecessary readmission. Further studies will focus on and explore ways to visualize care goals in the FTCP to prepare patients and staff more effectively concerning how to improve patient involvement. A patient version of FTCP with all the included care goals in the postoperative phase is planning to be developed by the research team. The overall aim of the patient version of FTCP is to visualize the care planning day by day. Continuous information about treatment and care goals during the care process may reduce confusion and insecurity about the postoperative care planning. Further studies will also explore mobile phone interventions to investigate the potential to provide an effective system for monitoring of patients in fast-track care with early discharge.

## Limitations

When interpreting the results, methodological limitations must be considered.

First, patients were only recruited from one university hospital, which might be a limitation. Patients from only one care setting were interviewed, and this may have influenced the findings and the transferability of results. Second, there is a risk of recall bias. When interviews were carried out up to 9 months postoperatively, patients may not remember all the details regarding their specific surgical care situation. The study aim was to explore patient experiences of involvement specifically related to fast-track surgical care, yet patients had other ongoing care contacts, for example, in oncology care. Furthermore, all participating patients experienced fast-track care and short hospital stays. The findings may have differed if patients experiencing standard care with longer hospital stays had also been interviewed about their experiences of involvement in care. It is also a possibility that patients with a preferable prognosis perceived a better sense of involvement, which may have related to the included patients’ social status.

## Conclusions

Patients who have had surgery for the liver, bile duct or pancreatic cancer need individualized care and continuous information about the FTCP during the care process. This may reduce anxiety and readmissions and may contribute to a safe environment at discharge. Individualized care and continuous information about treatment and care goals during the care process creates trust between patients and healthcare professionals and increases experiences of involvement.
